# Household Catastrophic Healthcare Expenditure and Impoverishment Due to Rotavirus Gastroenteritis Requiring Hospitalization in Malaysia

**DOI:** 10.1371/journal.pone.0125878

**Published:** 2015-05-05

**Authors:** Tharani Loganathan, Way-Seah Lee, Kok-Foo Lee, Mark Jit, Chiu-Wan Ng

**Affiliations:** 1 Department of Social and Preventive Medicine, University of Malaya, Kuala Lumpur, Malaysia; 2 Department of Paediatrics, University of Malaya, Kuala Lumpur, Malaysia; 3 University Malaya Paediatric and Child Health Research Group, Kuala Lumpur, Malaysia; 4 Paediatric Department, Hospital Sultanah Nur Zahirah, Kuala Terengganu, Malaysia; 5 Modeling and Economics Unit, Public Health England, London, United Kingdom; 6 Department of Infectious Disease Epidemiology, London School of Hygiene and Tropical Medicine, London, United Kingdom; National Institute for Viral Disease Control and Prevention, CDC, CHINA

## Abstract

**Background:**

While healthcare costs for rotavirus gastroenteritis requiring hospitalization may be burdensome on households in Malaysia, exploration on the distribution and catastrophic impact of these expenses on households are lacking.

**Objectives:**

We assessed the economic burden, levels and distribution of catastrophic healthcare expenditure, the poverty impact on households and inequities related to healthcare payments for acute gastroenteritis requiring hospitalization in Malaysia.

**Methods:**

A two-year prospective, hospital-based study was conducted from 2008 to 2010 in an urban (Kuala Lumpur) and rural (Kuala Terengganu) setting in Malaysia. All children under the age of 5 years admitted for acute gastroenteritis were included. Patients were screened for rotavirus and information on healthcare expenditure was obtained.

**Results:**

Of the 658 stool samples collected at both centers, 248 (38%) were positive for rotavirus. Direct and indirect costs incurred were significantly higher in Kuala Lumpur compared with Kuala Terengganu (US$222 Vs. US$45; p<0.001). The mean direct and indirect costs for rotavirus gastroenteritis consisted 20% of monthly household income in Kuala Lumpur, as compared with only 5% in Kuala Terengganu. Direct medical costs paid out-of-pocket caused 141 (33%) households in Kuala Lumpur to experience catastrophic expenditure and 11 (3%) households to incur poverty. However in Kuala Terengganu, only one household (0.5%) experienced catastrophic healthcare expenditure and none were impoverished. The lowest income quintile in Kuala Lumpur was more likely to experience catastrophic payments compared to the highest quintile (87% vs 8%). The concentration index for out-of-pocket healthcare payments was closer to zero at Kuala Lumpur (0.03) than at Kuala Terengganu (0.24).

**Conclusions:**

While urban households were wealthier, healthcare expenditure due to gastroenteritis had more catastrophic and poverty impact on the urban poor. Universal rotavirus vaccination would reduce both disease burden and health inequities in Malaysia.

## Introduction

Rotavirus (RV) gastroenteritis (GE) is the most common cause of childhood diarrhea worldwide. By the age of five, almost every child worldwide would have experienced at least one episode of RVGE [1]. In Malaysia, although mortality rates are low, RVGE causes significant morbidity. In 2005, it was estimated that by the age of five years, 1 in 37 children will visit a clinic, 1 in 61 children will be hospitalized, and 1 in 15,000 children will die as a result of RVGE [2]. The estimated median cost of providing inpatient care for an episode RVGE was US$212 (range US$69–881) in 2002 [3]. In 2009, the mean cost incurred by households for an episode of RVGE requiring hospitalization was estimated at US$194 (range US$47–738), constituting 26% of the average monthly household income [4]. Infant vaccines are available that are efficacious against RVGE, particularly in its severe forms [5–7], but they are not part of the universal vaccine schedule in Malaysia.

Although common, with adequate management RVGE is rarely fatal. Whilst out-of-pocket (OOP) healthcare expenditure incurred due to an acute diarrheal illness may not be large in magnitude, it may be catastrophic to some households. Healthcare expenditure paid OOP exceeding 10% of household income is widely considered to be catastrophic, as it can potentially disrupt household living standards [8]. Unexpected healthcare payments, incurred OOP may push families into poverty. Poor households are more vulnerable to sudden, unbudgeted OOP healthcare expenditures. The poor are likely to use a major portion of household income on basic necessities; hence OOP payments incurred to access care may deter the poor from seeking care [8–10]. The burden of OOP payment has a differential impact on poor and rich households, leading to income-related healthcare inequities. Hence a universal rotavirus vaccination program has the potential to provide financial risk protection by alleviating the economic burden due to childhood diarrhea, particularly for the poorest households. However, the magnitude and distribution of this burden for acute diarrheal illness has not been previously described in Malaysia, so the potential benefit of rotavirus vaccination in this respect is still not clear.

The present study was conducted to assess the direct and indirect costs incurred by households for an episode of RVGE requiring hospitalization, in order to inform decision-making around rotavirus vaccination in Malaysia. The study was based on patient data from two public hospitals, one serving a predominantly urban population and the other a relatively more rural population in Malaysia. The levels and distribution of catastrophic healthcare expenditure incurred, the poverty impact and income-related inequities of OOP healthcare expenditure was explored at both centers.

## Materials and Methods

### Population and Setting

A two-year, prospective, hospital-based study of RVGE was conducted from 1^st^ April 2008 to 31^st^ March 2010 at the pediatric departments of University of Malaya Medical Centre (UMMC), Kuala Lumpur (KL) and Hospital Sultanah Nur Zahirah (HSNZ), Kuala Terengganu (KT). This study was approved by the institutional ethics committees of both centers. (Approval numbers: 55-20-23-1025 and HSNZ.KT.100–23/9).

UMMC is a 1600-bedded, publicly funded, teaching hospital under the Ministry of Higher Education, located in Kuala Lumpur, the federal capital of Malaysia. It serves the mainly urban populations of Kuala Lumpur and the neighboring city of Petaling Jaya.

HSNZ is an 800-bedded, publicly funded, general hospital under the Ministry of Health, Malaysia. It serves as the referral hospital for state of Terengganu, on the east coast of Malaysia and caters to a predominantly rural population.

Both UMMC and HSNZ are tertiary medical hospitals, serving very different catchment populations. UMMC serves the population of Kuala Lumpur and Selangor. Kuala Lumpur is the capital city of Malaysia and more than 90% of the population of Selangor live in urban areas. In contrast, HSNZ serves as a referral center for the relatively rural state of Terengganu where about half of the population of the state live in rural areas[11].

### Inclusion Criteria

All children aged between one month and five years admitted for acute gastroenteritis (AGE) were considered eligible for the study. AGE was defined as acute diarrhea with frequent, loose stool, lasting less than 14 days prior to admission. Children with no diarrhea or diarrhea lasting more than 14 days were excluded. Children in immunocompromised states, with surgical conditions or systemic infections predisposing to diarrhea were also excluded.

Written informed consent was obtained from all parents or guardians prior to inclusion into the study. Information was collected by trained study personnel via face-to-face or telephone interviews. Fecal samples were obtained for RV detection.

### Data Collection

After informed consent was obtained, a standard, structured questionnaire was used to interview caregivers of patients. Details of the study protocol and results of viral genotyping have been published previously [12].

#### Stool Specimen Testing

Stool samples were tested for RV using a commercial kit, Premier Rotaclone (Meridian Biosciences, Cincinnati, Ohio). This enzyme immunoassay test has a sensitivity and specificity of 99% and is recommended for rotavirus surveillance by the World Health Organization [13].

#### Costing Components

Direct medical and non-medical costs incurred by the household were collected throughout the duration of diarrheal illness. All costs included were self-reported.

#### Direct Medical Costs

Direct medical costs are all OOP healthcare expenditure incurred, including consultation fees and medication costs incurred prior to admission, as well as hospital bills charged for the current and any other hospitalization within the same illness episode.

Full information on hospital bills paid by patients was collected at UMMC, however this information was not collected at HSNZ. As cost recovery at Ministry of Health facilities are less than 5% [14], hospital bills paid at HSNZ were assumed to be zero.

#### Direct Non-medical Costs

Direct non-medical costs include transportation costs, cost of extra diapers and special food. Transportation costs were additional expenditure related to travel while seeking care, like petrol, toll charges, parking fees and bus or taxi fares. The cost of extra diapers and special food as defined as expenditure above normal consumption due to illness.

#### Indirect Costs

Indirect costs were defined as the loss of productivity of parents for the duration of illness. Productivity loss of both parents were included. For working parents with self-reported monthly income and work-days missed, productivity loss was calculated by multiplying daily wage with days of work missed. As it was assumed that wage-earners worked a six-day week, the daily wage was calculated by dividing the self-reported monthly income by 26. Productivity loss for non-working parents or unpaid caregivers was not included.

#### Catastrophic Healthcare Expenditure

Catastrophic healthcare expenditure is defined here as OOP direct medical costs exceeding 10% of monthly household income [8].

#### Poverty Impact

We used the poverty line income for urban and rural regions in Peninsula Malaysia for 2009 from the Household Income and Basic Amenities survey [15]. A household is considered poor if its total monthly income falls below the poverty line.

The poverty headcount is the number of households with a total monthly household income below the poverty line. The poverty gap is defined as the income shortfall by which a poor household falls below the poverty line. The poverty headcount is a measure of incidence of poverty, while the poverty gap measures its intensity [8,16]. We explored both poverty headcounts and gaps before and after healthcare payments.

#### Concentration curve and concentration index

A concentration curve was plotted, representing the cumulative proportion OOP payments against the cumulative proportion of population ranked by increasing household income. The concentration index was defined as twice the area between the line of equality and the concentration curve. The line of equality is the diagonal line that represents no inequality in OOP payments by income groups. While the concentration curve is a measure of income related health inequities, the concentration index is used to quantify the degree of inequalities [8,17].

### Statistical Analysis

Statistical analysis was performed using SPSS version 20.0 (SPSS Inc., Chicago, Illinois, USA). Patients were divided into groups according to RV status: all cases of AGE, RVGE and non-RVGE.

Missing costs were assumed as zero. Handling of missing values of income is detailed in S1 File. Costs were inflated to 2013 price values [18]. Costs were reported as United States Dollar (US$). The exchange rate for 2013 of 3.15 was applied for currency conversion [19]. The Malaysian Poverty Line Income for 2009, stratified for urban and rural areas in Peninsular Malaysia was used. The poverty line for 2009 was converted using the 2009 World Bank exchange rate of 3.52 [15,19].

All costs, where appropriate, were presented as mean (± standard deviation, SD). Kolmogorov—Smirnov test was applied to test for normality of distribution. Independent sample t-test was used to test differences in means. Mann-Whitney U test was applied to test differences between groups when the assumption of normality was not met. A p-value of 0.05 was considered as statistically significant.

## Results

### General Findings

From the period of April 2008 to March 2010, a total of 800 children with AGE were admitted to the two study sites and 658 stool samples (82%) were tested for RV. Overall, 248 (37.7%) samples tested positive for RV. Of the 467 children admitted for AGE at UMMC, 385 (82%) had stool tested and of these 161 (41.8%) tested positive for RV. At HSNZ, of the 333 children admitted for AGE, 273 (82%) had stool samples tested, and 87 (31.9%) tested positive for RV.

At both centers, a hundred and fifty children (61%) with RVGE were aged two years or younger. While the median duration of diarrhea for an episode of RVGE was longer at UMMC compared to HSNZ (6 days vs. 5 days; p = 0.001), there was no difference in the median duration of admission at both centers (3 days; p = 0.264).

Patients with RVGE had increased frequency of fever and vomiting, compared with non-RVGE (p<0.05). Majority of children admitted with AGE at UMMC had moderate dehydration (92%), while 55% of children admitted at HSNZ had mild dehydration. Work days missed by both mothers and fathers of patients with RVGE were both significantly more at UMMC than at HSNZ (p<0.001) (Table 1). On average, households of patients at UMMC were wealthier than at HSNZ (US$1265 Vs. US$929; p<0.0001) (Table 2).

**Table 1:** Characteristics of children hospitalized for acute gastroenteritis and tested for rotavirus at UMMC, Kuala Lumpur and HSNZ, Kuala Terengganu.

Patient Characteristics	UMMC				HSNZ			
	All	RVGE	non-RVGE	p-	All	RVGE	non-RVGE	p-
	(n = 467)	(n = 161)	(n = 224)	value	(n = 333)	(n = 87)	(n = 186)	value
**Male (%)**	59%	57%	58%	0.86	61%	62%	63%	0.83
**Age, months (Median, IQR)**	17 (21)	19 (22)	15 (18)	0.00	17 (20)	18 (2)	13 (15)	0.00
**Degree of dehydration (%)**								
None or mild	32 (7%)	9 (6%)	14 (6%)		182 (55%)	37 (43%)	112 (61%)	
Moderate	428 (92%)	148 (93%)	209 (93%)		144 (43%)	49 (56%)	71 (38%)	
Severe	5 (1%)	3 (2%)	1 (0%)	0.39	5 (2%)	1 (1%)	2 (1%)	0.02
**Clinical Features**								
Vomiting (%)	429 (92%)	156 (97%)	197 (88%)	0.00	272 (82%)	81 (93%)	142 (76%)	0.00
Fever (%)	412 (88%)	154 (96%)	186 (83%)	0.00	273 (82%)	70 (81%)	123 (66%)	0.02
**Duration of diarrhea, days (median, IQR)**	5 (3)	6 (2)	5 (3)	0.04	5 (3)	5 (2)	5 (4)	0.91
**Duration of hospitalization, days (median, IQR)**	3 (2)	3 (1)	3 (2)	0.49	3 (2)	3 (1)	3 (2)	0.00
**Work Days Missed, days (mean ± SD)**								
Mother	3.0±1.5	3.1±1.4	2.9±1.7	0.42	0.9±1.6	1.1±1.5	0.7±1.4	0.08
Father	1.7±1.2	1.7±1.1	1.7±1.2	0.71	0.3±0.9	0.6±1.3	0.3±0.8	0.07

**NOTE**. IQR, interquartile range; mean (± standard deviation, SD); UMMC, University of Malaya Medical Centre; HSNZ, Hospital Sultanah Nur Zahirah;

All, acute gastroenteritis; RVGE, rotavirus gastroenteritis and non-RVGE, non-rotavirus gastroenteritis.

The p-value is a result of statistical comparison between rotavirus and non-rotavirus groups at each center. An alpha of <0.05 is significant.

**Table 2:** Costs incurred by households when children are hospitalized for acute gastroenteritis and tested for rotavirus at UMMC, Kuala Lumpur and HSNZ, Kuala Terengganu.

Cost Items	UMMC				HSNZ			
	All (n = 467)	RVGE (n = 161)	non-RVGE (n = 224)	p- value	All (n = 333)	RVGE (n = 87)	non-RVGE (n = 186)	p- value
	Mean ± SD	Mean ± SD	Mean ± SD		Mean ± SD	Mean ± SD	Mean ± SD	
Preadmission consultation	26 ± 73	20 ± 11	32 ± 105	0.14	4 ± 7	6 ± 8	4 ± 7	0.05
Consultation Cost	26 ± 73	20 ± 11	32 ± 105	0.15	3 ± 6	5 ± 7	3 ± 7	0.03
Medication Cost	0 ± 3	0 ± 1	0 ± 4	0.12	1 ± 2	1 ± 2	1 ± 3	0.79
Direct hospitalization payment	71 ± 57	71 ± 55	71 ± 55	0.97	0	0	0	
**Total direct medical costs**	97 ± 93	91 ± 56	103 ± 119	0.19	4 ± 7	6 ± 8	4 ± 7	0.05
Transport	20 ± 16	20 ± 10	21 ± 21	0.67	2 ± 3	3 ± 5	2 ± 2	0.24
Food expenses	1 ± 4	1 ± 2	1 ± 4	0.30	0 ± 1	0 ± 0	0 ± 2	0.03
Diapers	4 ± 3	5 ± 3	4 ± 3	0.11	2 ± 3	2 ± 2	2 ± 3	0.08
**Total direct non-medical costs**	26 ± 19	26 ± 12	26 ± 23	0.77	4 ± 5	4 ± 6	5 ± 4	0.82
**Total direct costs**	123 ± 98	116 ± 60	129 ± 125	0.24	8 ± 9	10 ± 10	8 ± 9	0.16
Productivity Loss Mother	50 ± 76	52 ± 80	47 ± 74	0.62	18 ± 42	24 ± 40	15 ± 45	0.19
Productivity Loss Father	50 ± 46	52 ± 53	48 ± 42	0.47	7 ± 24	9 ± 30	7 ± 23	0.66
**Total Indirect costs**	102 ± 92	107 ± 95	97 ± 91	0.35	26 ± 47	33 ± 47	22 ± 49	0.16
**Total Indirect and direct costs**	224 ± 154	222 ± 124	226 ± 178	0.82	35 ± 51	45 ± 32	32 ± 51	0.11
**Total monthly household income**	1265	1253	1241	0.94	929	1017	887	0.22

**NOTE**. All price values were inflated to 2013 Ringgit Malaysia (RM) and are reported in United States Dollar (US$), as mean (± standard deviation, SD). In 2013, 1 US$ was equivalent to 3.15 RM.

UMMC, University of Malaya Medical Centre; HSNZ, Hospital Sultanah Nur Zahirah.

All, acute gastroenteritis; RVGE, rotavirus gastroenteritis and non-RVGE, non-rotavirus gastroenteritis.

The p-value is a result of independent t-test between means of rotavirus and non-rotavirus groups at each center, alpha <0.05 is significant.

We found that the imputed and non-imputed datasets gave largely similar results. The imputed dataset was used for the estimation of costs (Table 2), to allow for the utilization of the full sample. Analysis for the catastrophic and poverty impact of out-of-pocket payments and the concentration index was done using the non-imputed datasets.

### Direct and indirect costs

There was no difference in direct and indirect costs between RVGE and non-RVGE groups at UMMC. At HSNZ, patients with RVGE had higher consultation costs for preadmission care compared to non-RVGE groups (US$5 Vs. US$3; p = 0.03), however other costs did not differ significantly between groups.

At UMMC, the average total direct costs for an episode of RVGE requiring hospital care was US$116 (SD ± US$60), of which 78% was due to direct medical costs. Seventy-eight percent of direct medical costs were contributed by direct hospitalization payment US$71 (SD ± US$55). The average direct and indirect costs were US$222 (SD ± US$124), of which productivity losses (48%) was a major component. The productivity loss experienced by both parents were US$107 (SD ± US$95).

All costs incurred by households at HSNZ were significantly lower than at UMMC (p<0.001). The mean direct costs for an episode of RVGE requiring admission was US$10 (SD ± US$10). As direct hospitalization charges at HSNZ were assumed to be zero, the main contributor for direct medical costs was preadmission consultations, US$6 (SD ± US$8). The average direct and indirect costs was US$45 (SD ± US$53), of which productivity losses for both parents accounted for 73% of spending (Table 2).

The mean direct and indirect costs incurred for RVGE were significantly higher in UMMC compared with HSNZ (US$222 Vs. US$45; p<0.001). The mean direct and indirect costs consisted 20% of monthly household income in UMMC, as compared with only 5% household income at HSNZ.

### Catastrophic healthcare expenditure

**Fig 1:**
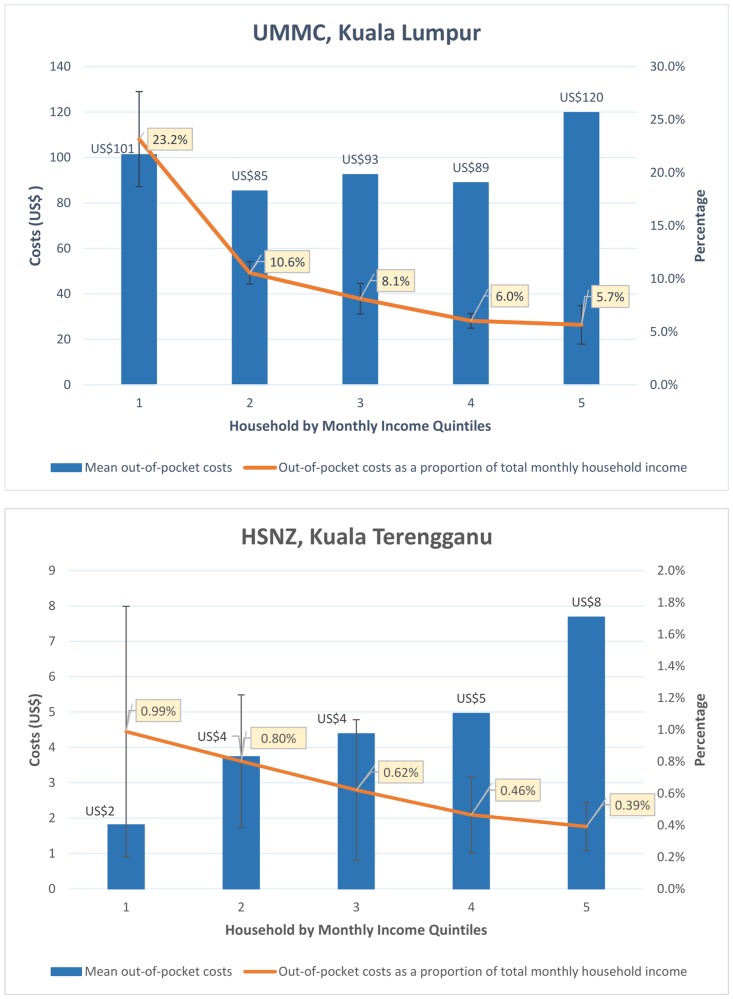
Mean out-of-pocket costs for acute gastroenteritis requiring hospitalization by income quintiles. **NOTE:** Error bars represent 95% confidence interval of out-of-pocket costs as a proportion of household income; Costs are in 2013 United States Dollars (US$); UMMC, University of Malaya Medical Centre; HSNZ, Hospital Sultanah Nur Zahirah.

On average, direct medical costs paid OOP for hospitalization of AGE at UMMC represented 11% of the total monthly household income. Catastrophic healthcare expenditure was experienced by a third of households at UMMC. Households in the highest income quintile had the highest average OOP expenditure (US$120; SD ± US$179), followed by households in the lowest income quintile (US$101; SD ± US$66). OOP expenditure as a proportion of monthly household income was significantly lower (5.7%) in the highest income quintile as compared to the lowest income quintile (23.2%) (p <0.001) (Fig 1). Eight percent of households in the highest income quintile experienced catastrophic healthcare payments, as compared to 86% in the lowest income quintile. When direct and indirect costs were considered 376 households at UMMC (88%) experienced healthcare payments of more than 10% of monthly household income.

**Fig 2:**
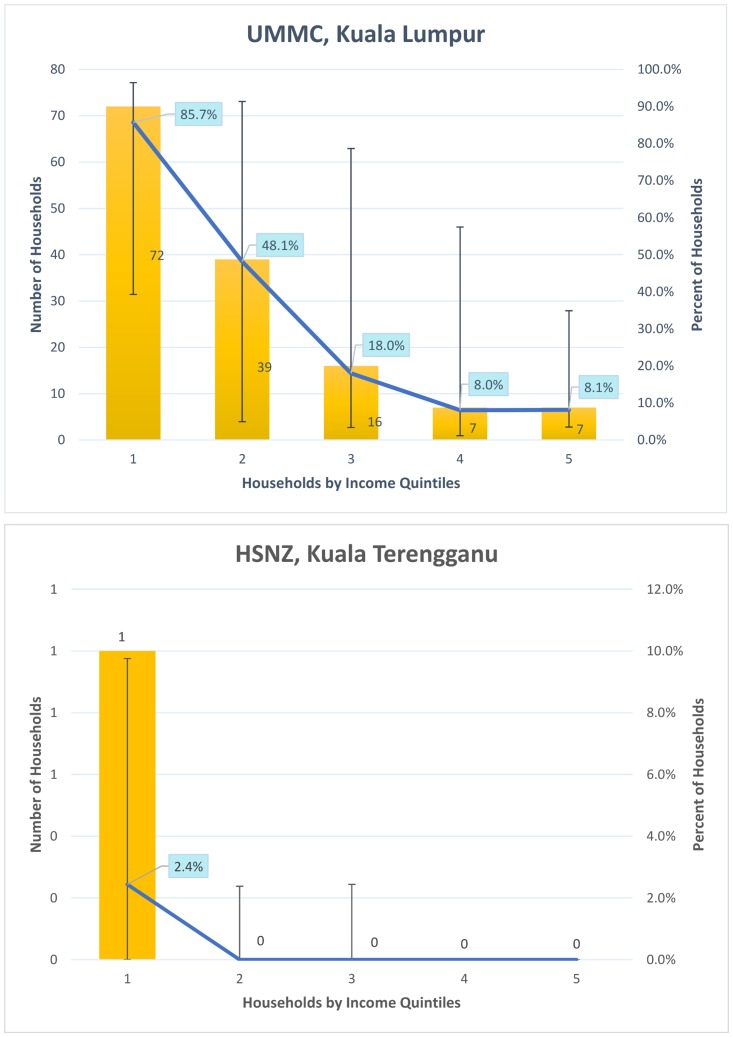
Households with catastrophic healthcare payments due to acute gastroenteritis requiring hospitalization. **Note**: Error bars represent range of results from varying thresholds of catastrophic payments from 5% to 20%. UMMC, University of Malaya Medical Centre; HSNZ, Hospital Sultanah Nur Zahirah.

In contrast at HSNZ, direct medical costs were less than one percent of the total monthly household income and only one household experienced catastrophic healthcare expenditure. Households in the highest income quintile had higher average expenditure OOP (US$8; SD ± US$11), compared to households in the lowest income quintile (US$2; SD ± US$5). This difference was statistically significant (p < 0.001) (Fig 2). When direct and indirect costs were considered, 19 households (9%) experienced healthcare expenditure of more than 10% of monthly household income.

### Poverty impact of healthcare payments

At UMMC, two households were below the poverty line prior to healthcare payments. Direct healthcare costs paid OOP displaced 11 households below the poverty line. When both direct and indirect healthcare costs were deducted from monthly incomes, 25 households were pushed below the poverty lines. At HSNZ, the pre-payment poverty headcount was 30. However, no households were impoverished after direct healthcare payments and only one household incurred poverty after direct and indirect costs. Post direct medical expenses the poverty gap widened by US$35.92 at UMMC and US$2.02 at HSNZ (Table 3).

**Table 3:** Poverty Impact of hospitalization for acute gastroenteritis at UMMC, Kuala Lumpur and HSNZ, Kuala Terengganu.

	UMMC	HSNZ
**Poverty Headcounts (no, %)**		
**Pre-payment**	2 (0%)	30 (14%)
**Post-payment**		
Post direct medical costs	13 (3%)	30 (14%)
Post direct costs	18 (4%)	30 (14%)
Post direct indirect costs	27 (6%)	31 (15%)
**Poverty Impact**		
Post direct medical costs	11 (3%)	0 (0%)
Post direct costs	16 (4%)	0 (0%)
Post direct indirect costs	25 (6%)	1 (0%)
**Poverty gaps (mean ± SD, US$)**		
**Pre-payment**	55.34 ± 63.13	46.10 ± 31.61
**Post-payment**		
Post direct medical costs	91.26 ± 87.31	48.12 ± 32.55
Post direct costs	78.65 ± 86.99	51.27 ± 33.39
Post direct indirect costs	72.98 ± 88.93	58.30 ± 33.21
**Poverty Impact**		
Post direct medical costs	35.92	2.02
Post direct costs	23.31	5.17
Post direct indirect costs	17.64	12.20

**NOTE**. All values are reported in 2009 United States Dollar (US$), as mean (± standard deviation, SD).

During the study period, 1 USD was equivalent to 3.36 Malaysian Ringgit (RM).

UMMC, University of Malaya Medical Centre; HSNZ, Hospital Sultanah Nur Zahirah.

Poverty line income in 2009 for urban regions, Kuala Lumpur US$ 219.03 and rural regions, Kuala Terengganu US$ 211.08 [15]

### Concentration curves and concentration index

**Fig 3:**
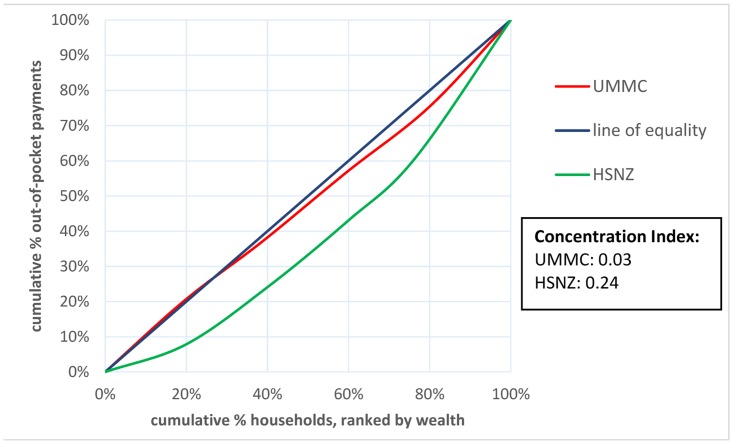
Concentration curves of out-of-pocket healthcare expenditure due to acute gastroenteritis requiring hospitalization. UMMC, University of Malaya Medical Centre; HSNZ, Hospital Sultanah Nur Zahirah.

The concentration curves for both centers fell below the line of equality indicating payments made OOP were concentrated among the rich. However, the concentration index of UMMC was closer to zero compared to that of HSNZ (0.03 Vs. 0.24). (Fig 3)

## Discussion

The present study shows that in Malaysia, the financial burden of AGE hospitalizations on households differs in urban and rural areas. Although households in the urban city of KL are wealthier than those in the rural community in KT (US$1265 Vs. US$929), all costs incurred in KL are significantly higher than in KT. On average, direct and indirect costs incurred by the households at KL for an episode of RVGE requiring hospitalization was US$222 (SD ± US$124), which consisted 20% of the total monthly household income. This is consistent with findings from a study conducted in the same setting in 2007 [4]. In contrast, direct and indirect costs due to an episode of RVGE requiring hospitalization at KT was a mere US$45 (SD ± US$53), or less than 6% of the total monthly household income.

We estimate that a third of households in KL experienced catastrophic healthcare payments. In addition, after incursion of direct and indirect healthcare expenses, 25 households (6%) were pushed into poverty. This catastrophic impact of healthcare expenses on households is unexpected in Malaysia, which is widely credited to have achieved Universal Health Coverage [20]. Malaysia has an extensive network of primary health clinics which provide free services for children, whilst admission to public hospitals are heavily subsidized [21]. Analysis of nationally representative household survey data show a low incidence of catastrophic healthcare payments in Malaysia, with only 2% of households spending more than 10% of total household budget on healthcare [22]. Also, the poverty headcount ratio for Malaysia in 2009 was estimated by the World Bank at 3.8% of the population [23].

However, it is surprising that at the rural center of KT, only one household incurred catastrophic payments due to hospitalization. While households at KT are less wealthy, healthcare expenses incurred were also significantly lower at KT (total direct medical costs for acute gastroenteritis; US$4 Vs. US$97; p<0.001). Low healthcare expenses incurred are a testimony to the success of the rural focus of public health services in Malaysia. Expenses are generally subsidized at all levels of care and in many forms [14]. For example, even non-urgent patients in rural settings may be transported from primary health clinics to hospital for admission via free ambulance services. Lower cost of living may explain lower direct non-medical costs like transport, food and diapers.

Interestingly, we found inequities in OOP expenditure along the income quintiles. In KL, households in the lowest income quintile paid more OOP for healthcare compared to all other households apart from those in the highest income quintile. Furthermore, households in the lowest income quintile were far more likely to experience catastrophic payments compared with those in the highest income quintile (86% Vs. 8%). This is important as it suggests that although the richest households paid more for healthcare, they were less likely to suffer change in living standards or incur debt due to healthcare expenses. In KT, although healthcare expenditure was higher in the highest income quintile as compared to the lowest income quintile (US$8 Vs. US$2; p<0.001), these costs had minimal catastrophic impact. The concentration curves suggest that while OOP healthcare payments at both centers were concentrated among the rich, interventions to alleviate OOP payments were more likely to be pro-poor in rural compared to urban regions. These findings are supported by the concentration index which is closer to zero at UMMC compared to that of HSNZ (0.03 Vs. 0.24). While the poor pay as much OOP as the rich in KL, the poor are largely protected from OOP healthcare payments at KT.

The Third National Health and Morbidity Survey showed that 47.6% of children under five years with acute diarrhea, first sought treatment at a private clinic [24]. In the present study, we found 79% (353) of children at KL, almost half of which belong to the two lowest income quintiles, had sought treatment at a private clinic prior to hospitalization.

The higher fee structure at UMMC compared to HSNZ and different payment collection mechanisms may explain the higher direct hospitalization payments at UMMC. UMMC is a teaching hospital under Ministry of Higher Education, and while fees are still subsidized, they are higher than other public hospitals under the Ministry of Health, Malaysia. As UMMC is the only public hospital in Petaling Jaya, a city with a population of almost a million, its fee structure impacts the poor. Our findings indicate an increased demand for affordable inpatient and outpatient healthcare services in the urban city of KL, which is unfulfilled by available public healthcare services.

Two rotavirus vaccines have been available internationally since 2006 [25] and although these vaccines are available commercially in the private market, Malaysia has yet to introduce the rotavirus vaccine universally. Our findings suggest that the economic burden of RVGE is particularly severe in poorer urban households, who may also be the least likely to purchase the vaccine privately.

Traditionally, evidence on disease burden and the availability of a safe and effective vaccine that is cost-effective and affordable, was enough to inform decision-making around vaccine introduction. Increasingly, the broader economic impact of vaccination including improvements in financial risk protection and equity, are important considerations [26–28]. The World Health Report 2013 states that ‘the goal of universal health coverage is to ensure that all people obtain necessary health services without risk of financial ruin or incurring poverty’ [29]. This current study demonstrates that the introduction of a rotavirus vaccine into a national immunization program may smooth the path towards universal health coverage by providing financial risk protection to households with the greatest need.

There are several limitations to this study. Both, income and costs obtained were self-reported, which may have led to under-reporting of wages or expenses being over-stated. To minimize bias we conducted multiple interviews at different time points using a structured questionnaire. Imputation techniques were used in handling missing values of household income.

The examination of the impact of healthcare expenditure on households should ideally be conducted using longitudinal data, to best estimate the extent of disruption of living standards due to health shocks. We studied the catastrophic and poverty impact of AGE using cross-sectional data from public hospitals in two different locations in Malaysia.

While it is usual to define healthcare expenditure as catastrophic when it exceeds a fraction of household income over a year, we compare OOP costs incurred for AGE with monthly income. Expenses incurred for acute diarrhea is short term and are unlikely to be repeated, as such we do not annualize costs or income. This must be interpreted with caution as expenditure incurred over a single month may not have the same implications on household welfare as expenses over a year, as coping mechanisms like savings and loans are easier to obtain on the short term.

As information on hospital charges actually paid by patients was unavailable at KT, we made the assumption that these charges were zero. Although it was possible to estimate the hospital bill from the daily ward charges and laboratory test charges, this would not accurately represent the OOP payments. While publicly provided healthcare services at Ministry of Health hospitals are highly subsidized, many patients do not settle hospital bills. Also, a large segment of patients are exempt from fees including school children, pregnant mothers, public sector employees and their dependents, people with disabilities and the socioeconomically disadvantaged.[14]. The Second National Health and Morbidity Survey which specifically looked at health seeking behavior and OOP healthcare payments in Malaysia found that 92.5% of respondents utilized government clinics and 66.4% used government hospitals for free. Furthermore, this survey demonstrated that the average hospital charges paid in the state of Terengganu was a third that of payments made at Kuala Lumpur [30]. At Kuala Lumpur and urban areas, public hospitals are more likely to collect fees as the population served is wealthier. In 2012, the mean average household income for Kuala Lumpur was RM 8,586 per month as compared to RM 3,967 per month in Terengganu and the national average income of RM 5,000 [31]. In 2006, unpaid bills amounted to RM 26.1 million or around 0.3% of the Ministry of Health budget [32].

Whilst, HSNZ is very representative of hospitals serving rural populations, UMMC may not be representative of all urban public hospitals in Malaysia. UMMC is unique in terms of its status as a teaching hospital and its location in Kuala Lumpur. As a teaching hospital under Ministry of Education, UMMC has a higher fee structure and payment collection mechanism than hospitals under the Ministry of Health. Also, UMMC is located in the most population dense city in Malaysia, and serves a more affluent population[11,31].

The information on direct hospitalization payments at UMMC was obtained through patient interviews. However, if a similar assumption of hospital charges being zero was made at UMMC, the differential impact of payments on household income across income quintiles will not be seen.

This study has several strengths. This is the first study of its kind to explore the catastrophic and poverty impact of AGE in Malaysia. By examining costs incurred by households for RVGE in urban and rural settings by income quintiles, we explored the potential distributional consequences of universal rotavirus vaccination in Malaysia.

## Conclusion

Although households in Kuala Lumpur are wealthier than those in Kuala Terengganu, it is paradoxical that poor urban households may be more vulnerable to the financial impact of healthcare expenditure. The introduction of a universal rotavirus vaccine as an effective preventive measure, may substantially benefit all families; not only by reducing the disease burden, but also by providing financial risk protection to households with the greatest need.

## Supporting Information

S1 File Text A. Handling of Missing Values. Table A. Missing Values for Total Household Income at UMMC, Kuala Lumpur and HSNZ, Kuala Terengganu. UMMC, University of Malaya Medical Centre; HSNZ, Hospital Sultanah Nur Zahirah. Table B. Poverty Impact of hospitalization for acute gastroenteritis at UMMC, Kuala Lumpur and HSNZ, Kuala Terengganu. Note: The imputed dataset uses pooled imputed values for Total Household Income. In the complete case analysis, cases with missing values for Total Household Income are deleted. All values are reported in 2009 United States Dollar (US$), as mean (± standard deviation, SD). During the study period, 1 USD was equivalent to 3.36 Malaysian Ringgit (RM). Poverty line income in 2009 for urban regions, Kuala Lumpur US$ 219.03 and rural regions, Kuala Terengganu US$ 211.08 [15]. UMMC, University of Malaya Medical Centre; HSNZ, Hospital Sultanah Nur Zahirah.
(DOCX)
