# Supplementary material for: Household Catastrophic Healthcare Expenditure and Impoverishment Due to Rotavirus Gastroenteritis Requiring Hospitalization in Malaysia
Source: PLoS One. 2015 May 5;10(5):e0125878. doi: 10.1371/journal.pone.0125878 (PMC4420491; doi:10.1371/journal.pone.0125878)
Supplement: S1 File — Text A. Handling of Missing Values. Table A. Missing Values for Total Household Income at UMMC, Kuala Lumpur and HSNZ, Kuala Terengganu. UMMC, University of Malaya Medical Centre; HSNZ, Hospital Sultanah Nur Zahirah. Table B. Poverty Impact of hospitalization for acute gastroenteritis at UMMC, Kuala Lumpur and HSNZ, Kuala Terengganu. Note: The imputed dataset uses pooled imputed values for Total Household Income. In the complete case analysis, cases with missing values for Total Household Income are deleted. All values are reported in 2009 United States Dollar (US$), as mean (± standard deviation, SD). During the study period, 1 USD was equivalent to 3.36 Malaysian Ringgit (RM). Poverty line income in 2009 for urban regions, Kuala Lumpur US$ 219.03 and rural regions, Kuala Terengganu US$ 211.08 [15]. UMMC, University of Malaya Medical Centre; HSNZ, Hospital Sultanah Nur Zahirah. (DOCX) [file pone.0125878.s001.docx]

# **Text A**

# **Handling of missing values**

The original questionnaire used for data collection did not collect information on monthly household income. These variables were included after the start of data collection, to allow for estimation of indirect costs and the impact of out-of-pocket payments on household income. As such, the initial 31 cases (7%) from University of Malaya Medical Centre (UMMC), Kuala Lumpur and 80 cases (24%) from Hospital Sultanah Nur Zahirah (HSNZ), Kuala Terengganu did not contain information on household income. Over the course of the study a further 9 cases (2%) from UMMC and 47 cases (14%) from HSNZ contained missing values for household income (Table A).

We examined patterns of missing values for total monthly income at both centers to inform appropriate measures to deal with missing values.

At UMMC, there was no difference in total direct costs between cases with missing total monthly income and those with no missing values (US$195 Vs. US$189; p=0.79). The Little’s (1988) MCAR test was non-significant (p=0.79) indicating that data was missing completely at random (MCAR)[[1](#_ENREF_1),[2](#_ENREF_2)].

At HSNZ, total direct costs were lower in those with missing values compared with cases with no missing values of total monthly income (US$6 Vs US$10; p<0.0001). Significant results of the Little’s MCAR test (p<0.0001), suggests that data was either missing at random (MAR) or not missing at random (NMAR)[[3](#_ENREF_3)].

## Discussion

In rural areas, some may rely on a traditional subsistence economy and may not receive a fixed wage (e.g. farmers, fisherman). This may explain non-disclosure of monthly income at Kuala Terengganu. While the possibility of NMAR if ignored may bias results, there is no consensus on the appropriate methods to be used to reduce bias [[4](#_ENREF_4)].

We used multiple imputations method with specifications to best deal with the missing values. In this study, information collected was mainly on clinical features and management of childhood diarrhea, health-seeking behavior and costs. Socio-demographic variables like parental education level, or housing status were not collected. Direct costs may be used to predict household income. Total household income is significantly correlated to direct costs. At HSNZ, the Pearson correlation coefficient is 0.2 (p=0.001). However, direct costs do not sufficiently explain the variation in total household income (F^2^= 0.05).

Multiple imputations was performed using SPSS version 20.0 (SPSS Inc., Chicago, Illinois, USA). Total direct costs was used as the independent (predictor) variable and total household income as the dependent (outcome) variable. Linear regression was used for the imputation model and a monotone imputation method was used. Five imputed datasets were generated and a pooled result was used for analysis.

We found that the imputed and non-imputed datasets gave largely similar results. We used the imputed dataset for the estimation of costs (Table 2 in the main text). This allowed utilization of the full sample for estimation of cost. As direct costs does not fully explain total household income, we used case-wise deletion for missing values of total household income for the rest of the analysis. Results of analysis for the catastrophic and poverty impact of out-of-pocket payments and the concentration index shown in the main text was done using the non-imputed datasets. Comparison of results of the poverty impact analysis between the imputed dataset and the non-imputed datasets are shown in Table B.

# **References**

1. Little RJ (1988) A test of missing completely at random for multivariate data with missing values. Journal of the American Statistical Association 83: 1198-1202.

2. Allison PD (2000) Missing data: Sage Thousand Oaks, CA.

3. Schlomer GL, Bauman S, Card NA (2010) Best practices for missing data management in counseling psychology. Journal of Counseling Psychology 57: 1.

4. Buhi ER, Goodson P, Neilands TB (2008) Out of sight, not out of mind: strategies for handling missing data. American journal of health behavior 32: 83-92.

5. Household Income and Basic Amenities Survey Report 2009. Department of Statistics, Malaysia. 97-99 p.

### Table A. Missing Values for Total Household Income at UMMC, Kuala Lumpur and HSNZ, Kuala Terengganu

|  | **UMMC** | | **HSNZ** | |
| --- | --- | --- | --- | --- |
| Total Sample | 467 | (100%) | 333 | (100%) |
| Household Income not collected | 31 | (7%) | 80 | (24%) |
| Missing values | 9 | (2%) | 47 | (14%) |
| Total Missing values (Household Income) | 40 | (9%) | 127 | (38%) |

UMMC, Universiti Malaya Medical Center; HSNZ, Hospital Sultanah Nur Zahirah

### Table B. Poverty Impact of hospitalization for acute gastroenteritis at UMMC, Kuala Lumpur and HSNZ, Kuala Terengganu.

|  | **UMMC  (n=467)** | | **UMMC   (n=427)** | | **HSNZ (n=333)** | | **HSNZ (n=207)** | |
| --- | --- | --- | --- | --- | --- | --- | --- | --- |
|  | **Imputed** | | **Non-Imputed** | | **Imputed** | | **Non-Imputed** | |
| **Poverty Headcounts (no, %)** |  |  |  |  |  |  |  |  |
| **Pre-payment** | 4 | (0.9%) | 2 | (0.5%) | 43 | (12.9%) | 30 | (14.5%) |
| **Post-payment** |  |  |  |  |  |  |  |  |
| Post direct medical costs | 16 | (3.4%) | 13 | (3.0%) | 43 | (12.9%) | 30 | (14.5%) |
| Post direct costs | 22 | (4.7%) | 18 | (4.2%) | 43 | (12.9%) | 30 | (14.5%) |
| Post direct and indirect costs | 26 | (5.6%) | 27 | (6.3%) | 44 | (13.2%) | 31 | (15.0%) |
| **Poverty Impact** |  |  |  |  |  |  |  |  |
| Post direct medical costs | 12 | (2.6%) | 11 | (2.6%) | 0 | (0.0%) | 0 | (0.0%) |
| Post direct costs | 18 | (3.9%) | 16 | (3.7%) | 0 | (0.0%) | 0 | (0.0%) |
| Post direct and indirect costs | 22 | (4.7%) | 25 | (5.9%) | 1 | (0.3%) | 1 | (0.5%) |
| **Poverty gaps (mean ± SD, US$)** |  |  |  |  |  |  |  |  |
| **Pre-payments** | 57.30 |  | 55.34 | ± 63.13 | 60.36 |  | 46.10 | ± 31.61 |
| **Post-payment** |  |  |  |  |  |  |  |  |
| Post direct medical costs | 92.78 |  | 91.26 | ± 87.31 | 62.21 |  | 48.12 | ± 32.55 |
| Post direct costs | 82.60 |  | 78.65 | ± 86.99 | 64.95 |  | 51.27 | ± 33.39 |
| Post direct and indirect costs | 65.08 |  | 72.98 | ± 88.93 | 69.62 |  | 58.30 | ± 33.21 |
| **Poverty Impact** |  |  |  |  |  |  |  |  |
| Post direct medical costs | 35.48 |  | 35.92 |  | 1.85 |  | 2.02 |  |
| Post direct costs | 25.30 |  | 23.31 |  | 4.59 |  | 5.17 |  |
| Post direct and indirect costs | 7.78 |  | 17.64 |  | 9.26 |  | 12.20 |  |

Note: The imputed dataset used imputed values for Total Household Income. In the non-imputed dataset, complete case analysis was done. Cases with missing values for Total Household Income are deleted.

All values are reported in 2009 United States Dollar (US$), as mean (± standard deviation, SD).

During the study period, 1 USD was equivalent to 3.36 Malaysian Ringgit (RM).

Poverty line income in 2009 for urban regions, Kuala Lumpur US$ 219.03 and rural regions, Kuala Terengganu US$ 211.08 [[5](#_ENREF_5)].

UMMC, University Malaya Medical Center; HSNZ, Hospital Sultanah Nur Zahirah.
